# Selections in Practical Medicine

**Published:** 1855-09

**Authors:** 


### SELECTIONS IN PRACTICAL MEDICINE

Dr. Gordon, Physician to the Hospital Hardwicke, recommends the use of chloroform in ℥ss doses to subdue nervous irritation in fevers. In a case in which "the pulse was 130 weak, raving continually; difficult to restrain; requiring straight waistcoat; constant talking, no sleep; tongue brown and dry in the centre; thirsty; eyes very congested, pupils dilated."

Under the continued use of chloroform in the above doses, he became much quieter and a still further use of the remedy put him to sleep from which he awoke refreshed and calm. From 5 P. M., to 8 A. M., the patient took 7½ drachms of chloroform.—*Ranking's H. Y. Abstract.*

*Hot douche in Typhoid Pneumonia.*—Dr. Jones, of Petersburgh, Virginia, used the hot douche in typhoid pneumonia where the patient was so far reduced as to lie on his back, knees drawn up, countenance sunken, pulse low, cerebral disturbance, tongue loaded, hurried respiration, cough, red tongue, &c. The patient was laid upon his face and five gallons of water, as hot as could be borne, were immediately thrown upon the spine, after which, he was wrapped in blankets and placed in bed, sleep followed. This was again repeated and the same results followed. Dovers powder grs. x. was also given and in a few days he was well.—*Ibid.*

*Cantharides and Strychnine in Incontinence of Urine in Children.*—Dr. Gross says that these remedies succeed when every thing else has failed. It must be continued until strangury is produced.—Ed.

*Iodide Potassium.*—This article gains power by combination with iodine itself. When the salt is adopted and the iodine admissible, they should be used in conjunction.—*Dublin Gazette.*

*Rheumatism.*—The treatment settled upon for this disease in the London Hospitals consists in the exhibition of the neutral salts. Some of the forms, as the acetate, tartrate or nitrate of potash. With the use of one of these remedies and prosecuted vigorously, the "six weeks" duration of the disease as the ordinary term of Watson is very materially shortened.—*American Journal from Med. Times & Gazette.*

*Injections of Water and Milk into the Peritoneal Cavity for the cure of Cholera.*—Dr. Herapath proposes a combination of these fluids to be injected in the peritoneal cavity, for the purpose of supplying to these tissues the serum they have lost, and as a combination of milk and water forms a compound similar to serum, and is supplied to these tissues to make up the deficiency.—*Dublin Gazette & Amer. Journal.*

*Pyrosis.*—Galic acid is recommended for the cure of this where there are no ulcers in the stomach. Water-brash, as it is termed, is soon relieved by its use.—*Asso. Jour. & Amer. Journal.*

*Fumes of Opium in Coryza.*—A few grains of powdered opium thrown upon a plate of metal heated, will give forth its fumes while the patient is to forcibly inhale it. This treatment is often successfull.—*Amer. Journal & Gazette Med.*

*Hiccough.*—Dr. Upsher says that "this symptom is the result of spasms of the diaphragm, and can be relieved by a bandage tightly applied around the lower part of the thorax, which relaxes the diaphragm and causes a cessation of the spasms. It may also prove of much assistance in relieving cases of chronic vomiting not dependent upon organic diseases of the stomach.—*Medical Counsellor.*

*Iodine and Nitrate of Silver.*—A correspondent in the Medical Counsellor, recommends for the treatment of erysipelas that the tincture of iodine should not be first used over the part—then to be immediately followed with the nitrate, the resulting compound being a dull white. This of course, is the iodide of silver. This course was recommended by Dr. Nutting, but the correspondent thinks that a patient's "leg" is no laboratory, and therefore, the preparation should be first made then applied.

Would the correspondent refrain from the use of soda in acidity of the stomach, lest he would make a *laboratory* of *that* organ?—Ed.

*Emmenagogue Properties of Chamomile Flowers.*—Dr. H. T. Brown maintains that the emmenagogue properties of these flowers are not appreciated. He uses the tincture prepared in the following manner. ℞ Chamomile flowers ℥ij, dilute alcohol Oj.—A teaspoonfull every three hours. A liniment of camphor, oil of turpentine and spirits ammonia, as an external application over the lumbar and pelvic regions. This treatment he thinks very favorably of in suppressed menstruation. For our part we are incredulous as to the powers of the so called emmenagogues. Chamomile flowers are regarded as tonic, and their virtues in his hands may have been found to reside in this property and not in any specific action. The cure, upon which he lays much stress, in order to exhibit forcibly the curative quality of this plant, may have been chlorotic, requiring a tonic and hence his success. We cannot but believe that if the diseased condition of the system, which suspends or even suppresses this discharge, can be overcome, then this, and other secretions will go on normally. If, to illustrate, the discharge is suspended by an intermittent attack, which last is removed by quinine, and after this the normal discharge takes place, is then the quinine in this instance an emmenagogue? Now on this principle we may largely multiply the class of emmenagogues. Lactation suspends the catamenia, but would chamomile or any other of the class of emmenagogues reproduce it? We think not. We have seen the menstrual discharge which had been suspended, reproduced under the use of iron, in cases where there was anæmia. The result in these cases, was but the effect of a cause, and that cause resided in the improvement in the general health by the tonic powers of the iron. Intussusception of the bowels produces obstinate constipation, one of its symptoms. But who, if he diagnosed the case correctly would rely upon cathartics, which would but aggravate the symptoms? Remove the spasm and thereby the obstruction, and the bowels will act *sua sponte*. In nineteen cases out of twenty of suppressio mensium, it is dependent upon some local or general disease of the system—a dependent circumstance and not a cause.—Ed.

*Tinitus Aurum.*—When it arises from scanty secretion of wax and a little deafness attending, a few drops of the spirit of nitric ether dropped into the ear or it may be used by moistening cotton with it and introducing it into the meatus.—*Ibid.*

*Nitric Acid in Hooping Cough.*—Dr. Gibb in his work on this disease, highly recommends that the acid be given as follows: ℞ Dilute Nitric Acid (London Pharm.) ʒj; Cardamum Comp. ʒiij; Syrup Simp. ℥iijss; Aqua ℥j. M. Dose a desert spoonful every hour or two to a child two years old.

(The introduction of the vaccine virus, which we have practiced for a number of years, into the arm is a remedy of as much potency as any other. Ed.)

*Gangrene of the Lung.*—Dr. Bowditch of the Mass. General Hospital cured a case of gangrene of the lung with *liquor sodæ chlorin* in ten drop doses, frequently repeated, anodyne inhalations and a generous diet.—Virg. Med. & Surg. Jour.

*Leibig's new broth for the Sick.*—Take a half pound of the flesh of a recently killed animal, (beef or the flesh of a fowl) and chop it fine and mix it well with a pound and eighth of distilled water, to which four drops of muriatic acid is added, with from half drachm to one drachm of muriate soda (common salt). After it has stood an hour the whole is thrown on a common hair sieve and the fluid is allowed to run off without pressure. The first that runs off is turbid and must be thrown back from time to time until it runs off clear. On to the fleshy residue in the sieve a half pound of distilled water is added in small portions. In this way a pound of fluid extract of meat is obtained, of a red color and an agreeable taste of broth. The sick can take a cup full at pleasure. It will not bear heat.—London Lancet.

(This has been tried by us and we find it a valuable article of diet and much liked by the patients for whom we have recommended it. It is composed of animal albumen and hæmatin, constituting the very best pabulum for the blood, so much required where there has been much exhaustion and those in which the vital constituents of the blood have been lost. A fine material out of which to manufacture red particles. Ed.)

*Lactate of Iron in Nervous Diseases.*—This article is used with antispasmodics in the following combination. ℞ Valerianate Zinc ʒij; Lactate of Iron ʒiss; Extract Belladona ʒss; Extract Valerion q. s. to make 60 pills. 2 are taken for the first two days and afterward gradually increased.—*Memphis Recorder.*

(We have found the above to be valuable in a case of epilepsy. Ed.)

*Solidified Milk.*—This is made by adding to 112 bls. of fresh milk, 28 lbs. white sugar, and a teaspoonfull of bi-carbonate of soda. It is then evaporated in a water-bath at a moderate temperature, being stirred and agitated all the while, but so moderately as to avoid churning. In three hours it assumes a pasty consistency, and by constant manipulation and warming, it is reduced to a rich, creamy-looking powder. It is then exposed to the air to cool, weighed into parcels of a pound each, and pressed into a brick-shaped tablet, which is covered with tin-foil. This will keep for any length of time, and may be grated and dissolved in water for use, answering all the purposes of ordinary milk, even to the making of butter. Our ships and steamers will find this solidified milk convenient and economical, and it may come into general use in cities. It is particularly convenient for use in sick-rooms and hospitals.—*Memphis Med. Recorder.*

*Tincture of Iodine with Chloroform in Inhalation.*—In the Bulletin de Therapeutique of Paris, M. Titon, calls attention to the perfect *solubility and volatiolity*, of iodine in chloroform. He says chloroform dissolves iodine even to complete saturation in the proportion of 20 to 100. Ten minutes after an inhalation of five minutes, the iodine was detected in the saliva, and in fifteen minutes in the urine. It may be breathed from a phial held to one of the nostrils for two, four, or ten minutes.

This is certainly a new and most efficient mode of administering iodine, decidedly one of the most valuable agents of our *materia medica.*—*Nashville Journal of Med. & Surgery.*

